# COVID-19 PICU guidelines: for high- and limited-resource settings

**DOI:** 10.1038/s41390-020-1053-9

**Published:** 2020-07-07

**Authors:** Saraswati Kache, Mohammod Jobayer Chisti, Felicity Gumbo, Ezekiel Mupere, Xia Zhi, Karthi Nallasamy, Satoshi Nakagawa, Jan Hau Lee, Matteo Di Nardo, Pedro de la Oliva, Chhavi Katyal, Kanwaljeet J. S. Anand, Daniela Carla de Souza, Vanessa Soares Lanziotti, Joseph Carcillo

**Affiliations:** 1 Department of Pediatrics, Stanford University School of Medicine, Palo Alto, CA USA; 2 Intensive Care Unit and Clinical Research, International Centre for Diarrhoeal Disease Research, Bangladesh (icddr,b), Dhaka, Bangladesh; 3 Department of Pediatrics and Child Health, College of Health Sciences University of Zimbabwe, Harare, Zimbabwe; 4 Department of Pediatrics and Child Health, School of Medicine College of Health Sciences, Makerere University, Kampala, Uganda; 5 Department of Pediatric Intensive Care Unit, Maternal and Child Health Hospital of Hubei Province, Wuhan City, Hubei Province China; 6 Department of Pediatrics, Postgraduate Institute of Medical Education and Research, Chandigarh, Punjab India; 7 Critical Care Medicine, National Center for Child Health & Development, Tokyo, Japan; 8 Children’s Intensive Care Unit, KK Women’s and Children’s Hospital, Duke-NUS Medical School, Singapore, Singapore; 9 Pediatric Intensive Care Unit, Children’s Hospital Bambino Gesù, Rome, Italy; 10 Pediatric Intensive Care Department, Hospital Universitario La Paz, Department of Pediatrics Medical School, Universidad Autónoma de Madrid, Madrid, Spain; 11 Pediatric Critical Care Medicine, The Children’s Hospital at Montefiore, Albert Einstein College of Medicine, Bronx, NY USA; 12 Department of Pediatrics, Anesthesiology, Perioperative & Pain Medicine, Stanford University School of Medicine, Palo Alto, CA USA; 13 Pediatric Intensive Care Unit, University of São Paulo & Hospital Sírio Libanês-, São Paulo, Brazil; 14 Pediatric Intensive Care Unit & Research and Education Division/Maternal and Child Health Postgraduate Program, Federal University of Rio De Janeiro, Rio De Janeiro, Brazil; 15 Departments of Critical Care Medicine and Pediatrics, University of Pittsburgh School of Medicine, Pittsburgh, PA USA

## Abstract

**Background:**

Fewer children than adults have been affected by the COVID-19 pandemic, and the clinical manifestations are distinct from those of adults. Some children particularly those with acute or chronic co-morbidities are likely to develop critical illness. Recently, a multisystem inflammatory syndrome (MIS-C) has been described in children with some of these patients requiring care in the pediatric ICU.

**Methods:**

An international collaboration was formed to review the available evidence and develop evidence-based guidelines for the care of critically ill children with SARS-CoV-2 infection. Where the evidence was lacking, those gaps were replaced with consensus-based guidelines.

**Results:**

This process has generated 44 recommendations related to pediatric COVID-19 patients presenting with respiratory distress or failure, sepsis or septic shock, cardiopulmonary arrest, MIS-C, those requiring adjuvant therapies, or ECMO. Evidence to explain the milder disease patterns in children and the potential to use repurposed anti-viral drugs, anti-inflammatory or anti-thrombotic therapies are also described.

**Conclusion:**

Brief summaries of pediatric SARS-CoV-2 infection in different regions of the world are included since few registries are capturing this data globally. These guidelines seek to harmonize the standards and strategies for intensive care that critically ill children with COVID-19 receive across the world.

**Impact:**

- At the time of publication, this is the latest evidence for managing critically ill children infected with SARS-CoV-2.
- Referring to these guidelines can decrease the morbidity and potentially the mortality of children effected by COVID-19 and its sequalae.
- These guidelines can be adapted to both high- and limited-resource settings.

## Introduction

In early December 2019, a novel coronavirus causing pneumonia was first reported in Wuhan, China and rapidly spread globally. Given the pathogenicity, high transmission, asymptomatic carrier rates, and global lack of human immunity to this virus, the World Health Organization pronounced a pandemic on March 11, 2020. Despite the aggressive nature of the Severe Acute Respiratory Syndrome Coronavirus 2 (SARS-CoV-2), children have been minimally affected by the disease.

At the time of publication, 8.4 million infections have been confirmed and 450,835 total deaths have been reported.^1^ The epidemiology of infection rates by age and mortality are difficult to identify globally, but pediatric cases have been relatively few. Two large studies, from China and the United States, have the best pediatric data to date. The largest pediatric review reported 2143 children from China (34.1% laboratory confirmed and 65.9% suspected cases as of February 7, 2020), 5.6% children had severe respiratory disease, 0.6% developed acute respiratory distress syndrome (ARDS), and 1 died.^2^ In contrast, 18.5% of adults had developed severe or critical illness.^2^ In the United States, pediatric patients made up only 1.7% of total cases from February 12 to April 2; only 20% of these required hospitalization, 2% required intensive care unit (ICU) admissions and three died.^3^ Both studies noted that patients under 1 year of age were at greatest risk of hospitalization and males accounted for 57% of cases.^2,3^ For those patients where data were recorded, nearly a quarter of patients had an underlying medical condition.^3^ Factors associated with worse outcomes included younger age, underlying pulmonary conditions, and immunocompromised states.^4^ The median age of children in China was 7 years^2,5^ and in the United States was 11 years,^3^ suggesting regional differences in age distribution. Most children therefore are asymptomatic or have mild to moderate symptoms and the case fatality rate (CFR, 0–0.2%) in children is much lower as compared to adults (CFR 2.3%).^6^

Common signs and symptoms among pediatric patients include fever, cough, myalgias, sore throat, rhinorrhea, headache, shortness of breath, diarrhea, vomiting, tachypnea, and tachycardia.^2,3,5^ Gastrointestinal symptoms of vomiting and diarrhea appear to be more common in pediatric patients as compared to adults.^4,6,7^ Adult patients that progress to severe disease often develop dyspnea 8 days after disease onset and within 48 h can progress to ARDS and multisystem organ dysfunction.^8^ Other findings in critically ill patients include shock, encephalopathy, myocardial dysfunction, heart failure, coagulopathy, and acute renal injury.^2^ Patients with severe COVID-19 appear to have a cytokine storm profile similar to hemophagocytic lymphohistiocytosis.^7^ Recently, a Multisystem Inflammatory Syndrome in Children (MIS-C) has also been described and patient presentations range from mild inflammation to severe shock with multiorgan involvement.

The progression of this pandemic in the ensuing months is unclear and pediatric intensivists globally will need to be aware of signs, symptoms, and treatments for COVID-19. The objective of this international collaboration is to present management strategies for critically ill pediatric patients infected with COVID-19 in both high- and limited-resource settings.

### Evaluation of minimal disease burden in children

Why are children relatively protected from COVID-19 illnesses? Several theories have been proposed although scientific knowledge in this regard is still evolving.

SARS-CoV-2 spike protein binds to angiotensin-converting enzyme II (ACE2) receptor to infect cells.^9^ ACE2 is expressed in the type II alveolar epithelial cells in the lungs, myocardial cells, esophageal, and ileocolic epithelial cells, and may also play a role in the coagulation system.^10^ The organotypic expression of this receptor may explain the presenting signs and symptoms of patients with COVID-19. An interplay between the renin-angiotensin system (RAS) and various proteins may increase or decrease inflammation in lung injury.^10–13^ Prevalent use of ACE-inhibitors (ACEi) or angiotensin-receptor blockers (ARB) in adults increases ACE2 expression, which may allow SARS-CoV-2 entry into type II alveolar cells leading to worsening lung disease.^10,11^ Conversely, other studies suggest that ACEi and ARBs diminish Angiotensin II production which in turn attenuates lung inflammation and injury.^10,11^ Relative absence of ACE2 expression in children is currently touted as one theory why children are protected from severe COVID-19 disease.^14^ Animal models demonstrate that levels of ACE2 receptor vary with development and by sex.^15,16^ Children from 4 to 18 years of age also have higher ACE activity than adults,^17^ which may be protective.

Age-based differences in immunity and inflammatory responses across the lifespan could also explain these outcomes. Specifically, lymphopenia occurred in only 3% of pediatric patients^18^ but 80% of critically ill adults.^8^ Similar inferences about inflammatory markers such as C-reactive protein or procalcitonin are more difficult to draw at this time. Lymphopenia may contribute to the inability to clear SARS-CoV-2 infection.

Another hypothesis is the antioxidant effects of melatonin to attenuate the inflammatory injury occurring from viral infections.^19,20^ Although melatonin may not decrease viral replication, it indirectly regulates ACE2 and may impact the ability of SARS-CoV-2 to enter, infect, and replicate.^19^ Ultimately, melatonin may prolong a patient’s survival allowing the individual’s immune system to eradicate the virus. Similar to ICU supportive care, melatonin affords the patient time to recover. Interestingly, the nighttime concentrations of melatonin in children are significantly higher than adults.^20^ Thus, the direct antioxidant effects of melatonin coupled with its effects on ACE2 receptors may play a protective effect in pediatric patients.

Understanding why children are relatively protected from severe SARS-CoV-2 infection may help us develop targeted drugs for symptomatic adult and pediatric patients.

## Methods

Pediatric intensivists with many years of clinical experience and academic backgrounds were invited from across the globe to develop these guidelines. Authors from both high- and limited-resource settings and all regions of the world (except Oceania) are represented. All contributing authors were assigned to various recommendation sections along with writing the specific section for their global region. Given the paucity of data in pediatric patients to date, a systematic review with meta-analysis of multiple studies and case series could not be used to evaluate the strength of each recommendation. The authors therefore have identified relevant adult and pediatric studies along with management guidelines from other leading organizations, e.g. the World Health Organization, and have extracted the best current evidence and guidelines to support their recommendations. Each guideline is followed by a *Strong, Weak, Best practice*, or *Insufficient evidence* recommendation (Table 1) and the rationale for each recommendation is included in the body of the manuscript. Recommendations could not be formulated using the Evidence to Decision framework (EtD) due to the paucity of relevant evidence regarding COVID-19 in pediatric patients and the rapidly changing landscape of this disease.

**Table 1:** Recommendations for the management of children infected with COVID-19.

Recommendation	Strength
Ventilation	
1. In children with COVID-19 infection and hypoxemia, begin supplemental oxygen therapy by low-flow nasal cannula when oxygen saturations (SpO_2_) are <90%. If the patient continues with hypoxemia, oxygen delivery via a face mask with reservoir bag should be initiated.	Strong
2. Children with COVID-19 that remain with increased work of breathing and hypoxemia should be escalated to high flow nasal cannula (HFNC) if available. Patients with progressive respiratory distress or where HFNC is unavailable can be escalated to noninvasive positive pressure ventilation (NIPPV), bubble continuous positive airway pressure (bCPAP) or bi-level positive airway pressure (BiPAP).	Strong
3. Children with COVID-19 failing NIPPV should be escalated to mechanical ventilation.	Strong
4. In children with COVID-19 requiring intubation, the procedure should be done by a trained and skilled health-care provider.	Strong
5. Children with COVID-19 requiring intubation should be intubated with a cuffed endotracheal tube.	Strong
6. For children with COVID-19 requiring intubation, use of video laryngoscopy should be considered for intubation.	Weak
7. Personal protective equipment (PPE) should be worn for intubation and extubation of all children with COVID-19.	Strong
8. For children with COVID-19 requiring mechanical ventilation, tidal volumes should be limited to 6 ml/kg.	Weak
9. For children with COVID-19 requiring mechanical ventilation, positive end expiratory pressure (PEEP) titration should be individualized to each patient and their phase of ARDS.	Weak
10. For children with COVID-19 requiring mechanical ventilation, prone position should be considered in patients with ARDS and severe hypoxemia.	Weak
11. For children with COVID-19 requiring mechanical ventilation with refractory hypoxemia, use of Inhaled nitric oxide is not recommended.	Insufficient evidence
12. For children with COVID-19 requiring mechanical ventilation, high-frequency oscillatory ventilation (HFOV) is not recommended for routine application but may be considered in select cases.	Insufficient evidence
Hemodynamic support	
13. For children with COVID-19 and shock admitted to health systems with PICU availability (ventilatory support and access to vasoactive amines), administer bolus fluids, 10–20 ml/kg per bolus up to 40–60 ml/kg, over the first hour of resuscitation.	Weak
14. For children with COVID-19 and shock admitted to health systems without PICU availability (no ventilatory support and access to vasoactive amines):	
a. *Patients without hypotension*, no fluid bolus should be administered, and maintenance fluids should be initiated.	Strong
b. *Patients with hypotension*, administer bolus fluids, 10–20 ml/kg per bolus up to 40 ml/kg, over the first hour of resuscitation.	Weak
15. In children with COVID-19 and shock, crystalloid solutions should be administered, instead of colloids, for the initial fluid resuscitation. Specifically, we recommend use of balanced solutions over 0.9% saline.	Weak
16. In children with COVID-19 and shock, age-appropriate mean arterial pressure (MAP) should be targeted. In settings where accurate MAPs cannot be easily obtained, systolic blood pressure is an acceptable option.	Strong
17. In children with COVID-19 and shock, consider the use of advanced hemodynamic variables, when available (measurements of cardiac index, systemic vascular resistance, and central venous oxygen saturation); these along with clinical variables at the bedside can guide resuscitation.	Weak
18. In children with COVID-19 and shock, in addition to clinical evaluation, trends in blood lactate levels can help guide resuscitation.	Weak
19. In children with COVID-19 and shock, epinephrine or norepinephrine should be administered, instead of dopamine. Diluted solution can be initiated through a peripheral intravenous catheter if central venous access is not available.	Best practice
20. In children with COVID-19 and shock who need high doses of catecholamines, consider initiating vasopressin.	Best practice
21. In children with COVID-19 and shock, recommendations regarding the use of inodilators cannot be made. But in clinical practice, inodilators such as milrinone, dobutamine or levosimendan could be used when there are signs of tissue hypoperfusion and cardiac dysfunction, despite high doses of catecholamines.	Best practice
22. In children with COVID-19 and refractory shock, consider anti-inflammatory doses of glucocorticoids.	Insufficient evidence
23. In a pediatric patient with COVID-19 and severe disease, a thorough cardiac evaluation should be conducted including an EKG, echocardiography and cardiac biomarker levels (troponin, CK and CK MB).	Strong
24. Glucocorticoid anti-inflammatory therapy and intravenous immunoglobulin (IVIG) are potential suggested treatments for children with COVID-19-related myocarditis.	Insufficient evidence
Adjuvant therapy	
25. Consider anticoagulation therapy in children with COVID-19 with: a. Mild risk of venous thromboembolism (VTE) (i.e. those with indwelling central or peripheral central venous catheters or severely ill with no hyperinflammatory status and with no risk of thrombosis), consider: i. Subcutaneous enoxaparin < 2 months: 0.75 mg/kg/dose q12 h; ≥2 months: 0.5 mg/kg/dose q12 h ii. Anti-Xa factor target: 0.3–0.5 IU/ml b. Children with COVID-19 with high risk of VTE (i.e. critically ill, hyperinflammatory state—C-reactive protein > 150 mg/l, D-dimer > 1500 ng/ml, IL-6 > 100 pg/ml, ferritin > 500 ng/ml, or past history of thromboembolic events) consider: i. Subcutaneous enoxaparin < 2 months: 1.5 mg/kg/dose q12 h; ≥2 months: 1 mg/kg/dose q12 h ii. Anti-Xa factor target: 0.5–1 IU/ml	Weak
26. In critically ill children with COVID-19, thrombocytopenia-associated multiple organ failure (TAMOF: platelet counts of less than 100 × 10^9^/L and two or more failing organs) and acquired ADAMTS-13 deficiency could indicate a thrombotic microangiopathic process. For such patients, therapeutic plasma exchange may be beneficial in controlling the hyperinflammatory and thrombotic state and reversing organ dysfunction.	Weak
27. For critically ill children with COVID-19, we suggest convalescent plasma treatment be offered only within a research framework or on a compassionate basis.	Insufficient evidence
28. For children with COVID-19 admitted to the PICU, initiate enteral nutrition for patients with no contraindications, but parenteral nutrition need not be initiated in the first 7 days of PICU admission.	Weak
29. For children with COVID-19 that develop fluid overload or renal dysfunction and are unresponsive to diuretic therapy consider renal replacement therapy.	Weak
CPR/Resuscitation	
30. Limit the number of personnel in the room for pediatric patients with COVID-19 that require cardiopulmonary resuscitation.	Best practice
31. For COVID-19 pediatric patients that have arrested follow standard cardiac arrest guidelines for CPR ratios, and treatments.	Best practice
32. For intubated patients, recommendations include: a. Increase the FIO_2_ to 1.0. b. Change mode to Pressure Control Ventilation and limit pressure as needed to generate adequate chest rise. c. Adjusting the trigger to Off will prevent the ventilator from auto-triggering with chest compressions and may prevent hyperventilation. d. Adjust respiratory rate to 10/min for adults and children. e. Adjust PEEP level to balance lung volumes and venous return. f. If return of spontaneous circulation is achieved, set ventilator settings as appropriate to patient’s clinical condition	Best practice
33. Patients in prone position at time of cardiac arrest without an advanced airway should be placed supine. Intubated patients, however, can remain prone and CPR can be initiated while the patient is prone.	Best practice
34. For children with COVID-19 that require PICU admission, address the goals of care with the parents or proxy as life-sustaining therapies are being escalated.	Best practice
ECMO considerations	
35. ECMO should be considered in COVID-19-infected pediatric patients to manage ARDS and/or cardiac failure (myocarditis, arrhythmias, pulmonary embolism).	Strong
36. Strict selection criteria for both Veno-Arterial (VA) and Veno-Venous (VV) ECMO should be applied in order to utilize ECMO for those patients most likely to benefit from the procedure.	Strong
Pediatric Multisystem Inflammatory Syndrome—Children (MIS-C)	
37. Supportive management and advanced monitoring should be provided (see “Hemodynamic support“ section).	
38. Early EKG and echography for cardiac function and coronary flow should be completed.	Best practice
39. Imaging studies should include chest X-ray, abdominal ultrasound, chest CT.	Best practice
40. A multidisciplinary team, including Intensive Care, Cardiology, Infectious Disease and Rheumatology, should manage these patients.	Best practice
41. Laboratory evaluation should include testing for SARS-CoV-2, biomarkers for inflammation, and multiorgan system dysfunction.	Best practice
42. Initiate empirical antibiotics until bacterial sepsis, staphylococcal or streptococcal syndrome are excluded.	Best practice
43. Medications to consider for management of MIS-C include solumedrol, IVIG, anticoagulants (see “Adjuvant therapy” section), and biologics (e.g. Anakinra, IL-6 inhibitors) and should be based on patient’s severity of illness using a multidisciplinary approach.	Insufficient evidence
44. For patients that specifically meet Kawasaki disease criteria (classic or atypical), administer IVIG (2 g/kg) and high-dose aspirin (50 mg/kg).	Strong

## Recommendations

### Respiratory support

#### Clinical presentation

##### Mild respiratory disease

Pediatric patients presenting with mild illness can be discharged and monitored at home. The family should be advised to strictly adhere with quarantine guidelines, use acetaminophen or paracetamol as a first-line agent for fever control (see “Pharmacologic treatment” section for further discussion), and to monitor the progression of clinical symptoms.

##### Severe respiratory disease

WHO defines severe respiratory distress in children presenting with tachypnea and any of the following: central cyanosis or hypoxemia (oxygen saturation [SpO_2_] < 90%), grunting, inability to breastfeed or drink, lethargy, unconsciousness, or convulsions.^21^ Age-specific tachypnea definitions are respiratory rate ≥ 60/min in children < 2 months of age, ≥50/min in children 2–11 months of age, ≥40/min in children 1–5 years of age.^21^

##### Acute respiratory distress syndrome

Worsening respiratory symptoms 1 week after disease onset due to SARS-CoV-2 with new opacities on chest imaging not explained by cardiac failure or volume overload and with a partial pressure of oxygen (PaO_2_) to fraction of inspired oxygen (FiO_2_) ratio ≤ 300 mmHg, or an SpO_2_/FiO_2_ < 264 during noninvasive ventilation, or an oxygenation index > 4, or an oxygen saturation index (OSI) > 5 during invasive mechanical ventilation are concerning for ARDS. Oxygen saturation index can be calculated based on pulse oximeter information without blood gas measurements; therefore, it can be used in the resource-limited environment and may facilitate early recognition of ARDS. Pediatric-specific and resource-limited setting definitions have also been defined previously.^22,23^

#### Treatment recommendations for oxygen therapy and noninvasive mechanical support

1. In children with COVID-19 infection and hypoxemia, begin supplemental oxygen therapy by low-flow nasal cannula when oxygen saturations (SpO_2_) are <90%.^24^ If the patient continues with hypoxemia, oxygen delivery via a face mask with reservoir bag should be initiated (*Strong recommendation*).
2. Children with COVID-19 that remain with increased work of breathing and hypoxemia should be escalated to High Flow Nasal Cannula (HFNC) if available. Patients with progressive respiratory distress, or where HFNC is unavailable, can be escalated to noninvasive positive pressure ventilation (NIPPV), bubble continuous positive airway pressure (bCPAP) or bi-level positive airway pressure (BiPAP) (*Strong recommendation*).

#### Rationale

Patients with respiratory distress should be treated when oxygen saturations are <90%.^25^ HFNC or NIPPV are safe and efficacious modes of respiratory support in pediatric patients and may provide adequate respiratory support to prevent the need for invasive mechanical ventilation. At a time when there is a global shortage of ventilators, particularly in resource-limited settings, alternative methods of respiratory support such as bCPAP should be considered. WHO recommends the use of bubble CPAP for treatment of COVID-19-infected children with hypoxemia, severe pneumonia and/or ARDS based on trials in Bangladesh and Ghana.^26–29^ These studies demonstrate improved outcomes in pediatric patients treated with locally developed bCPAP devices. Thus, in situations where mechanical ventilation might not be available, bCPAP may be used for newborns and children with severe hypoxemia, and may more readily be available in resource-limited settings.^26^ A recent systematic review revealed that the use of HFNC and bCPAP in children with severe pneumonia decreased the need for intubation and mechanical ventilation; younger children in particular had a greater benefit from the use of bCPAP.^29^ It is imperative that patients treated with either HFNC or NIPPV should be closely monitored for clinical deterioration. Finally, since all forms of respiratory support are at risk of aerosolization, health-care providers must assure airborne precautions and proper personal protective equipment is used.

#### Treatment recommendations for invasive mechanical ventilation

3. Children with COVID-19 failing NIPPV should be escalated to invasive mechanical ventilation (*Strong recommendation*).
4. In children with COVID-19 requiring intubation, the procedure should be done by a trained and skilled health-care provider (*Strong recommendation*).
5. Children with COVID-19 requiring intubation should be intubated with a cuffed endotracheal tube (*Strong recommendation*).
6. For children with COVID-19 requiring intubation, use of video laryngoscopy should be considered for intubation (*Weak recommendation*).
7. Personal protective equipment (PPE) should be worn for intubation and extubation of all children with COVID-19 (*Strong recommendation*).
8. For children with COVID-19 requiring mechanical ventilation, tidal volumes should be limited to 6 ml/kg (*Weak recommendation*).
9. For children with COVID-19 requiring mechanical ventilation, positive end expiratory pressure (PEEP) titration should be individualized to each patient and their phase of ARDS (*Weak recommendation*).
10. For children with COVID-19 requiring mechanical ventilation, prone position should be considered in patients with ARDS and severe hypoxemia (*Weak recommendation*).
11. For children with COVID-19 requiring mechanical ventilation with refractory hypoxemia, use of inhaled nitric oxide is not recommended (*Insufficient evidence*).
12. For children with COVID-19 requiring mechanical ventilation, high-frequency oscillatory ventilation (HFOV) is not recommended for routine application but may be considered in select cases (*Insufficient evidence*).

#### Rationale

Patients with progressive hypoxemia, altered mental status, or continued increased work of breathing on NIPPV should undergo endotracheal intubation (e.g. evolving ARDS). Multiple intubation attempts can result in poorer outcomes for the patient^30^ and place the provider at greater risk of exposure to COVID-19; therefore, intubation by the most-skilled provider is recommended. Before endotracheal intubation, bag-mask respiratory assistance may become an aerosol-generating procedure; therefore, rapid sequence intubation (RSI) using muscle relaxant is recommended. Pre-oxygenation with 100% oxygen (unless contraindicated, e.g. cyanotic heart lesion patients) is recommended to minimize hypoxia with RSI; children are at great risk of hypoxia due to a smaller functional residual capacity. Video laryngoscopy, if available, can be used to increase the distance between the patient and provider.^31^ Once the patient is intubated, the endotracheal tube cuff must be inflated immediately to prevent aerosolization. Endotracheal intubation is an aerosol-generating procedure; the provider therefore requires full PPE to protect himself/herself. PPE includes disposable fluid-resistant long-sleeved gowns, goggles or disposable full-face shields, and N95 masks. If available, a powered air-purifying respirator (PAPR) can be used in place of an N95 mask. Disposable operating room caps should be worn to reduce contaminating hairs by droplets.^32^

In order to provide further protection of health-care workers, other procedures have also been introduced, including an acryl intubation box,^33^ plastic drapes,^30^ as well as mask-over-tube technique.^34^ Use of hydrophobic viral filters placed at the ventilator outlets and inline closed suction are suggested to minimize the risk of transmission. Extubation is also an aerosol-generating procedure; therefore, PPE must be worn.

General management guidelines or protocols^35^ for mechanical ventilation in ARDS can be applied to the COVID-19 patients with respiratory failure. A low tidal volume, 4−6 ml/kg for ideal body weight, lung protective strategy is usually recommended.^35^ However, larger tidal volumes are frequently used in pediatric intensive care units (PICU) around the world^36^; therefore, it is advised to continue to use tidal volumes familiar to each institution. A meta-analysis of observational studies in children did not demonstrate a survival benefit by reducing tidal volumes^37^ and a retrospective analysis of ARDS patients at a single center also did not show a relation between tidal volumes and outcomes.^38^

Regarding the PEEP level, the experience in adult COVID-19 patients in Italy does not advise the use of higher PEEP routinely^39^ which varies from previous recommendations of PEEP use in ARDS.^35^ In the early phase of respiratory failure with COVID-19, the majority of patients exhibit critical hypoxemia due to increased intrapulmonary shunt fraction; however, the lung compliance is relatively maintained.^39^ In this situation, applying high PEEP may have detrimental effects, and applying relatively low PEEP and accepting lower oxygen saturations (80s to 90s) may be advised if the patient has single organ failure of the lungs. In the later phase, the pathophysiology may change to typical ARDS^39^ requiring a higher PEEP. Individualized titration of PEEP is recommended and the patient’s hemodynamics must be monitored closely with increasing PEEP.

Studies have demonstrated that prone positioning can improve oxygenation.^40^ However, this strategy should be implemented only by teams that routinely prone patients. Inhaled nitric oxide may improve oxygenation, but no studies to date have demonstrated a survival benefit.^41^ High-frequency oscillatory ventilation (HFOV) can be considered for refractory respiratory failure, but no clear data exist to support its use in COVID-19 patients. Hence, conventional mechanical ventilation using individualized PEEP is advised^42^; patients with preserved compliance may not require high PEEP whereas those with low lung compliance will benefit from more PEEP.

Finally, although not listed as a recommendation, a conservative fluid strategy should be used in patients with ARDS if the patient’s hemodynamics will allow fluid restriction.

If available, extracorporeal membrane oxygenation (ECMO) may be considered in patients with continued severe hypoxemia despite maximal support (see “ECMO considerations” section).

### Hemodynamic support

#### Clinical presentation

Clinical presentation of children with COVID-19 can range from tachycardia to classic signs of shock and heart failure. The presence of tachycardia varies from 6 to 42%.^5,43^

Reports in adult patients indicate that patients with myocardial injury may have arrhythmias and myocarditis. The presence of myocardial injury and increased cardiac biomarkers are associated with worse outcomes with a tenfold increase in mortality.^44^ Cardiac injury can be detected by elevated cardiac biomarkers, such as troponin, creatine kinase (CK), and its isoenzyme MB, electrocardiogram (EKG) and echocardiographic abnormalities. Cardiac enlargement can be evidenced in chest X-ray or CT scan.^45,46^ Pediatric patients at risk of developing myocarditis include those with comorbidities, such as heart disease, or those who progress to the severe hyperinflammatory phase.

#### Treatment recommendations for shock

13. For children with COVID-19 and shock admitted to health systems with PICU availability (ventilatory support and access to vasoactive amines), administer bolus fluids, 10–20 ml/kg per bolus up to 40–60 ml/kg, over the first hour of resuscitation^47^ (*Weak recommendation*).
14. For children with COVID-19 and shock admitted to health systems without PICU availability (no ventilatory support and access to vasoactive amines)^47^:
  *Patients without hypotension*, no fluid bolus should be administered and maintenance fluids should be initiated (*Strong recommendation*).
  *Patients with hypotension*, administer bolus fluids, 10–20 ml/kg per bolus up to 40 ml/kg, over the first hour of resuscitation (*Weak recommendation*).
15. In children with COVID-19 and shock, crystalloid solutions should be administered, instead of colloids, for the initial fluid resuscitation. Specifically, we recommend use of balanced solutions over 0.9% saline (*Weak recommendation*).
16. In children with COVID-19 and shock, age-appropriate mean arterial pressure (MAP) should be targeted. In settings where accurate MAPs cannot be easily obtained, systolic blood pressure is an acceptable option (*Strong recommendation*).
17. In children with COVID-19 and shock, consider the use of advanced hemodynamic variables, when available (measurements of cardiac index, systemic vascular resistance, and central venous oxygen saturation); these along with clinical variables at the bedside can guide resuscitation (*Weak recommendation*).
18. In children with COVID-19 and shock, in addition to clinical evaluation, trends in blood lactate levels can help guide resuscitation (*Weak recommendation*).
19. In children with COVID-19 and shock, epinephrine or norepinephrine should be administered, instead of dopamine. Diluted solution can be initiated through a peripheral intravenous catheter if central venous access is not available (*Best practice recommendation*).
20. In children with COVID-19 and shock who need high doses of catecholamines, consider initiating vasopressin (*Best practice recommendation*).
21. In children with COVID-19 and shock, recommendations regarding the use of inodilators cannot be made. But in clinical practice, inodilators such as milrinone, dobutamine or levosimendan could be used when there are signs of tissue hypoperfusion and cardiac dysfunction, despite high doses of catecholamines (*Best Practice recommendation*).
22. In children with COVID-19 and refractory shock, consider anti-inflammatory doses of glucocorticoids (*Insufficient evidence*).
23. In a pediatric patient with COVID-19 and severe disease, a thorough cardiac evaluation should be conducted including an EKG, echocardiography, and cardiac biomarker levels (troponin, CK and CK MB) (*Strong recommendation*).
24. Glucocorticoid anti-inflammatory therapy and intravenous immunoglobulin (IVIG) are potential suggested treatments for children with COVID-19-related myocarditis (*Insufficient evidence*).

#### Rationale

The need for bolus fluid administration should be guided by frequent clinical assessments (heart rate, blood pressure, capillary refill, level of consciousness, urine output), serial serum lactate levels, and advanced hemodynamic monitoring when available. In the presence of fluid overload, (signs of pulmonary edema and/or hepatomegaly), fluid resuscitation should be reassessed. In settings with advanced supportive care availability, recent studies show no difference in mortality between the restrictive and permissive fluid resuscitation strategies.^47–50^ The FEAST trial done in Africa evaluating fluid resuscitation in limited-resource settings demonstrates the need to be cautious of aggressive fluid resuscitation.^51^

Studies demonstrate no outcome benefits for patients resuscitated with crystalloid over colloid solutions. Crystalloid solutions are recommended based on lower cost, greater availability, and the transfusion risk associated with colloid use.

Although blood lactate is not considered a direct measure of tissue perfusion, high lactate is associated with worse outcomes in children.^52^ Additionally, adult data demonstrated significantly lower mortality and improvement in other outcomes when resuscitation was guided by serial lactate measurements.^53–56^ Persistently elevated blood lactate levels may indicate incomplete hemodynamic resuscitation and should trigger efforts to restore adequate tissue perfusion and establish hemodynamic stability.

For inotropic support, epinephrine or norepinephrine should be chosen as the first-line vasoactive infusion guided by clinical preferences, individual patient physiology, and local system factors. Both have inotropic and vasopressor effects and are effective in treating children with fluid refractory shock. Epinephrine should be considered as the first-line agent in patients with myocardial dysfunction and norepinephrine for patients with low systemic vascular resistance, as recommended in Surviving Sepsis Campaign (SSC) guidelines.^47^ Institutional practices and the availability of vasopressin should determine if and when it can be initiated. Although there are no RCTs for inodilators in children with shock, they should be considered in patients with myocarditis or low cardiac output syndrome. Glucocorticoids could also be considered for patients with refractory shock (see “Pharmacologic treatment” section for further discussion).

For patients with signs of myocarditis, in addition to supportive treatment, some anecdotal reports in adult patients with COVID-19 and myocarditis suggest IVIG therapy.^57,58^

### Adjuvant therapy

#### Clinical presentation

In this section, we discuss several important supportive therapies that may be applicable in critically ill children with COVID-19. Many of these therapies are described in critically ill adults but their description in the pediatric population remains limited. These therapies include the management of the prothrombotic state that occurs frequently in adult patients with COVID-19, convalescent plasma infusion, nutritional therapy, and renal replacement therapies.

#### Treatment recommendations

25. Consider anticoagulation therapy for children with COVID-19 (*Weak recommendation*):
  -  Mild risk of venous thromboembolism (VTE) (i.e. those with indwelling central or peripheral central venous catheters^59,60^ or severely ill with no hyperinflammatory status and with no risk of thrombosis), consider:
    i.  Subcutaneous enoxaparin < 2 months: 0.75 mg/kg/dose q12 h; ≥2 months: 0.5 mg/kg/dose q12 h
    ii.  Anti-Xa factor target: 0.3–0.5 IU/ml
  -  Children with COVID-19 with high risk of VTE (i.e. critically ill, hyperinflammatory state—C-reactive protein > 150 mg/l, D-dimer > 1500 ng/ml, IL-6 > 100 pg/ml, ferritin >500 ng/ml, or past history of thromboembolic events) consider:
    i.  Subcutaneous enoxaparin < 2 months: 1.5 mg/kg/dose q12 h; ≥2 months: 1 mg/kg/dose q12 h
    ii.  Anti-Xa factor target: 0.5–1 IU/ml
26. In critically ill children with COVID-19, thrombocytopenia-associated multiple organ failure (TAMOF: platelet counts of less than 100 × 10^9^/l and two or more failing organs), and acquired ADAMTS-13 deficiency could indicate a thrombotic microangiopathic process. For such patients, therapeutic plasma exchange may be beneficial in controlling the hyperinflammatory and thrombotic state and reversing organ dysfunction^61,62^ (*Weak recommendation*).
27. For critically ill children with COVID-19, we suggest convalescent plasma treatment be offered only within a research framework or on a compassionate basis (*Insufficient evidence*).
28. For children with COVID-19 admitted to the PICU, initiate enteral nutrition for patients with no contraindications, but parenteral nutrition should not be initiated in the first 7 days of PICU admission (*Weak recommendation*).
29. For children with COVID-19 that develop fluid overload or renal dysfunction and are unresponsive to diuretic therapy consider renal replacement therapy (*Weak recommendation*).

#### Rationale

High rates of venous thromboembolism (e.g., central line thrombosis and vascular occlusive events such as ischemic limbs and strokes) have been observed in critically ill COVID-19 adult patients.^63–68^ Although COVID-19 pediatric patients have not experienced thrombotic complications, the coagulation abnormalities reported are consistent with a hyperinflammatory prothrombotic state: elevated levels of D-dimer, C-reactive protein, procalcitonin, IL-6, and thrombocytopenia.^18,69–71^ A hyperinflammatory response to the infection may be the main pathogenic mechanism involved.^72,73^ In adults with COVID-19 infection, heparin seemed to decrease mortality in those patients with high D-dimer.^74,75^ Laboratory tests to consider in patients with thrombotic complications include: platelet count, PTT, fibrinogen, antithrombin III, ADAMTS-13, IL-6, D-dimer, C-reactive protein, procalcitonin, ferritin, plasma-free hemoglobin, schistocytes, LDH, haptoglobin, and thromboelastography (TEG).

Therapeutic plasma exchange has been proposed for patients with fulminant COVID-19 infection since it offers benefit on multiple levels by removing inflammatory cytokines, stabilizing endothelial membranes, and resetting the hypercoagulable state.^76^ However, use of plasma exchange therapy in children with COVID-19 has not been reported to date.

In previous pandemics (e.g., SARS, H1N1 2009, MEWRS-CoV), convalescent plasma treatments were utilized as part of the management strategies.^77–79^ Because convalescent plasma can potentially suppress viremia in COVID-19,^80^ there is increasing description of convalescent plasma treatment, albeit mainly as case reports and case series.

To date, there are no specific nutrition guidelines for critically ill children with COVID-19. The Society of Critical Care Medicine together with American Society for Parenteral and Enteral Nutrition have provided some guidance on the nutrition therapy for critically ill adult patients.^81^

Current available evidence from critically ill adults with COVID-19 found that acute kidney injury (AKI) is not a common phenomenon^82^ and no recommendations were made in the adult COVID-19 surviving sepsis guidelines.^83^ To date, there are no specific data on the incidence of AKI in critically ill children with COVID-19.

### Cardiopulmonary resuscitation (CPR)

#### Clinical presentation

In pediatric patients infected with COVID-19 that are pulseless due to asystole, pulseless electrical activity (PEA), ventricular fibrillation, or pulseless ventricular tachycardia, the following recommendations should be considered.

#### Treatment recommendations

30. Limit the number of personnel in the room for pediatric patients with COVID-19 that require cardiopulmonary resuscitation (*Best practice recommendation*).
31. For COVID-19 pediatric patients with cardiac arrest, follow the standard cardiac arrest guidelines for CPR ratios, and treatments (*Best practice recommendation*).
32. For intubated patients, recommendations include (*Best practice recommendation*):
  -  Increase the fractional inspired oxygen (FiO_2_) to 1.0.
  -  Change mode to Pressure Control Ventilation and limit pressure as needed to generate adequate chest rise.
  -  Adjusting the trigger to Off will prevent the ventilator from auto-triggering with chest compressions and may prevent hyperventilation.
  -  Adjust respiratory rate to 10/min for adults and children.
  -  Adjust PEEP level to balance lung volumes and venous return.
  -  If return of spontaneous circulation is achieved, set ventilator settings as appropriate to patient’s clinical condition.
33. Patients in prone position at the time of cardiac arrest without an advanced airway should be placed supine. Intubated patients however can remain prone and CPR can be initiated while the patient is prone (*Best practice recommendation*).
34. For children with COVID-19 that require PICU admission, address the goals of care with the parents or proxy as life-sustaining therapies are being escalated (*Best practice recommendation*).

#### Rationale

The American Heart Association (AHA) has modified conventional guidelines^84^ and released the following general principles when administering CPR to patients with suspected COVID-19. The guidelines have been modified to protect health-care providers and reduce COVID-19 exposure. All rescuers must don PPE to guard against both airborne and droplet particles. Limiting personnel in the room is recommended. If available, use of mechanical CPR devices on young adolescents instead of manual chest compressions is recommended to protect rescuers.

To lower the aerosolization risk of COVID-19, patients should be intubated with a cuffed endotracheal tube and connected to a ventilator with an inline suction catheter and a high efficiency particulate air (HEPA) filter in the path of exhaled gas. If possible, an HEPA filter should be attached to all manual or mechanical ventilation devices (see “Ventilation” section regarding intubation).

For patients that are already intubated at the time of arrest, they should remain on the ventilator with the HEPA filter to minimize aerosolization. General ventilator principles are to optimize oxygenation and venous return while minimizing hyperinflation.^85^

Patients who are prone at the time of arrest without an advanced airway, the recommendation is to place them supine and continue resuscitation. While the effectiveness of CPR in the prone position is not completely known, for proned patients with an advanced airway, avoid turning the patient to the supine position unless able to do so without risk of equipment disconnections and aerosolization. Instead, consider placing defibrillator pads in the anterior-posterior position and provide CPR with the patient remaining prone with hands in the standard position over the T7/10 vertebral bodies.^86^

Consider the appropriateness of starting and continuing CPR in COVID-19 patients. It is reasonable to consider age, comorbidities, and severity of illness in determining the appropriateness of resuscitation and balance the likelihood of success against the risk to rescuers and patients from whom resources are being diverted. It is recommended to address the goals of care with parents (or proxy) prior to an arrest situation.

### ECMO considerations

#### Clinical presentation

In PICU patients with SARS-CoV-2 that develop refractory hypoxemia or hypotension despite maximal medical support, ECMO may be considered.

#### Treatment recommendations

35. ECMO should be considered in COVID-19-infected pediatric patients to manage ARDS and/or cardiac failure (myocarditis, arrhythmias, pulmonary embolism) (*Strong recommendation*).
36. Strict selection criteria for both Veno-Arterial (VA) and Veno-Venous (VV) ECMO should be applied in order to utilize ECMO for those patients most likely to benefit from the procedure (*Strong recommendation*).

#### Rationale

The WHO,^25^ the Society of Critical Care Medicine^83^ and Extracorporeal Life Support Organization^87^ have all recommended the potential use of ECMO in severe cases of COVID-19. However, at the time of submitting this publication, specific indications and contraindications for its use in COVID-19 are scarce and outcome data derived from China are not encouraging.^88^ However, preliminary positive experiences in South Korea and Japan suggest the use of ECMO to manage SARS-CoV-2-induced ARDS and/or cardiac failure (myocarditis, arrhythmias, pulmonary embolism) unresponsive to maximal medical therapy.^89^

Initiation and patient selection for both VA and VV ECMO should not deviate from the existing guidelines.^90^ Early ECMO deployment is suggested to avoid progression to multiorgan failure. Older age, presence of more than two comorbidities, immunocompromised state (lymphopenia) are not absolute contraindications; however, each case should be evaluated on an individual basis.^91,92^ In a pandemic, ECMO should be initiated only at an experienced center, but even in such centers the availability of equipment and human capital should be considered prior to deployment of such a resource-intensive intervention. Extracorporeal cardiopulmonary resuscitation (eCPR) should not be initiated in less experienced centers or centers without an existing eCPR program prior to the pandemic. As disease burden increases, selection criteria for both respiratory and cardiac ECMO will become more stringent in order to utilize ECMO for those patients most likely to benefit. Given the pathophysiology of SARS-CoV-2 in humans, children may have better outcomes than adult patients and therefore may be a population in which ECMO resources should be invested.

### Pediatric multisystem inflammatory syndrome—children (MISC-C)

#### Clinical presentation/case definition

Potential presenting signs for MIS-C are fever, fatigue, altered mental status, neck pain, respiratory difficulty, chest pain, vomiting, diarrhea, abdominal pain, rash, and conjunctivitis. Specifically, case definition of MIS-C per the Center for Disease Control include the following^93^:

- Patient < 21 years of age presenting with fever (≥38.0 °C for ≥ 24 h, or report of subjective fever lasting ≥24 h), altered inflammations markers (C-reactive protein, erythrocyte sedimentation rate, fibrinogen, procalcitonin, D-dimer, ferritin, lactic acid dehydrogenase, or IL-6, elevated neutrophils, reduced lymphocytes, low albumin), and clinically severe illness requiring hospitalization, with two or more organs being involved (cardiac, renal, respiratory, hematologic, gastrointestinal, dermatologic or neurological); AND
- No other possible diagnoses (such as bacterial sepsis, staphylococcal or streptococcal syndrome); AND
- Recent or current SARS-CoV-2 infection, positive reverse-transcriptase polymerase chain reaction (RT-PCR) or serology test, or COVID-19 exposure in a period of 4 weeks prior to symptoms onset.

It must be noted that patient’s severity of illness in the hospital may range from mild to severe. Multisystem Inflammatory Syndrome should be considered in any pediatric death with evidence of SARS-CoV-2 infection.

#### Treatment recommendations for patients with concern for MIS-C

37. Supportive management and advanced monitoring should be provided (see “Hemodynamic support” section).
38. Early EKG and echography for cardiac function and coronary flow should be completed (*Best practice recommendation*).
39. Imaging studies should include chest X-ray, abdominal ultrasound, chest CT (*Best practice recommendation*).
40. A multidisciplinary team, including Intensive Care, Cardiology, Infectious Disease and Rheumatology, should manage these patients (*Best practice recommendation*).
41. Laboratory evaluation should include testing for SARS-CoV-2, biomarkers for inflammation, and multiorgan system dysfunction (*Best practice recommendation*).
42. Initiate empirical antibiotics until bacterial sepsis, staphylococcal or streptococcal syndrome are excluded (*Best practice recommendation*).
43. Medications to consider for management of MIS-C include solumedrol, IVIG, anticoagulants (see “Adjuvant therapy” section), and biologics (e.g. Anakinra, IL-6 inhibitors) and should be based on patient’s severity of illness using a multidisciplinary approach (*Insufficient evidence*).
44. For patients that specifically meet Kawasaki disease criteria (classic or atypical), administer IVIG (2 g/kg) and high-dose aspirin (50 mg/kg) (*Strong recommendation*).

#### Rationale

Since the initial National Health Service alert in England on April 26, multiple case reports have described children presenting with Kawasaki disease-like features and hyperinflammatory shock.^94–96^ At the Italian epicenter of SARS-CoV-2, a 30-fold increase was noted of children presenting with severe Kawasaki-like disease,^97^ and New York city reported on 15 children (ages 2–15 years) that required hospitalization (April 16−May 4) for multisystem inflammatory syndrome. Soon after, a Health Alert from the CDC^93^ described this disease entity and labeled it MIS-C. These patients test positive for COVID-19 either by RT-PCR or serology suggesting a pathogenic link between SARS-CoV-2 and MIS-C. The potential mechanism for the inflammation and mediated organ damage is unclear at present.

Given the novelty of MIS-C, treatment recommendations and best practices are based on treatments used in other aggressive inflammatory disease processes. Institutions with high number of MIS-C cases have developed institutional protocols regarding drug dosing and regimens based on patient’s severity of illness, but no definitive data exist to date.

### Pharmacologic treatments

There is insufficient evidence to date to make any definitive recommendations on pharmacologic therapies for pediatric patients with SARS-CoV-2. Children are protected from COVID-19 in part because they have low ACE2, which are entry points for the COVID-19 spike protein to attach and enter the host. Treatment of fever with acetaminophen or paracetamol is prudent since the impact of nonsteroidal anti-inflammatory drugs (NSAIDs) on children with COVID-19 is unclear and being investigated. Because children account for <0.1% of deaths from SARS-CoV-2, information on COVID-19-specific pharmacologic treatments is extrapolated from the adult Chinese, French, and Italian experience. The first report from Wuhan, China on hospitalized adult patients with SARS-CoV-2 revealed hyperferritinemic sepsis^98^ in those who died. Fever developed on day 1, sepsis on day 10, ICU admission on day 12 (for ARDS), and death at 19 days. Hyperferritinemia on days 4 and 7 predicted mortality long before development of sepsis and ICU admission. Progression to death was marked by increasing ferritin, C-reactive protein, IL-6, LDH, and D-dimer levels. Pharmacologic approach presently under study in adults^99^ has been conceived to target a first phase of viral infection (antiviral strategies), and then a second phase of hyperinflammatory (corticosteroid and biologic strategies) and prothrombotic (antithrombotic strategies) host responses (Table 2).

**Table 2:** Pharmacologic therapies.

Pharmacologic therapies under study in adults for COVID-19	Optimal clinical timing
Antiviral	Before day 4 of symptoms
Remdesivir	
Lopinavir/ritinovir	
Immune enhancement	
Interferons	Days 4−7 of symptoms
Convalescent plasma	In hospital
Anti-inflammatory	Days 4–7 of symptoms when ferritin > 500
Corticosteroids (Methylprednisone)	
Interleukin 1 Receptor Antagonist Protein	
Interleukin 6 inhibitor	

#### Repurposed antiviral strategies for SAR-CoV-2

Repurposed antiviral agents include remdesivir (ebola), and lopinavir/ritinovir (HIV); and repurposed immune-enhancing therapies include type I interferons (multiple sclerosis), and passive immunization with convalescent plasma. Effectiveness of these antiviral agents is best when given early in the course of infection before day 4 of symptoms. Use of immune-enhancing interferons is also likely to have benefit when given before need of hospitalization. For practical reasons, convalescent plasma should be reserved for hospitalized patients. Unfortunately, the ongoing adult clinical trials investigating the potential effectiveness of these therapies mostly do not adhere to these biologically plausible timelines. It is unlikely that their full benefit, if present, will be revealed in the present ongoing adult studies. Pediatric dosing experience exists for use of lopinavir/ritonavir (HIV), and type 1 interferons (chronic active hepatitis).

#### Repurposed anti-inflammatory strategies

These strategies can be considered when there is bedside evidence for hyperinflammation. Adult studies are presently using some combination of ferritin levels > 500, LDH > 300, and D-dimers > 1000 to identify eligible patients. Anti-inflammatory agents with minimal immune-suppressive effects that are under study include corticosteroids (methylprednisone), interleukin 1 receptor antagonists, and interleukin 6 receptor inhibitors. Use of these agents are more likely to be beneficial if started when hyperferritinemia occurs, which started at day 4 in the Wuhan experience. Therefore, monitoring ferritin should begin at day 4 of illness and the therapies are given before day 7 if hyperferritinemia occurs, and optimally before requiring intensive care at day 12. It is important to note that the hyperinflammatory response occurs in a minority of COVID-19 patients but is associated with the highest mortality. Unlike antivirals, these anti-inflammatory therapies should be reserved only for patients with hyperferritinemic inflammation. Pediatric dosing experience exists with corticosteroids and interleukin 1 receptor antagonist protein (Systemic Juvenile Arthritis induced Macrophage Activation Syndrome) and interleukin 6 inhibitors (CAR T-cell therapy induced Cytokine Releasing Syndrome). These therapies can and should also be considered for patients with MIS-C.

## Regional experience

Several databases have recently begun tracking pediatric patients affected by SARS-CoV-2 globally, but the authors gathered data from best available resources for their respective regions.

### Africa

Due to limited testing and difficulty in tracking cases, the data from Africa are limited. But data that can be identified are listed here. In Uganda, as of April 23, 63 cases have been confirmed, 9 of which are children ≤19 years of age.^100^ It is unclear how many of those patients may have required hospitalization.

In Zimbabwe, 203 total cases have been confirmed and the number of pediatric cases is unknown as they did not require ICU admission as of June 2. With widespread antibody screening now being initiated in the country, more accurate numbers of infected persons should be available. Based on National Institute for Communicable Diseases (NICD) data from South Africa, as of April 19, 219 persons under 20 years of age were infected with COVID-19 but PICU admission data were not available (communication with Dr. Felicity Gumbo).

### Asia

As of April 17, 82,000 cases of COVID-19 cases were confirmed in China. Wuhan city has reported 50,333 cases with 600 of those being pediatric patients.^101,102^ Only a small proportion of the pediatric cases required PICU admission.^71^ Many patients that developed critical illness had underlying pathologies, e.g. congenital heart disease, malnutrition.^71^ From the initial experience described in China, pediatric patients were mainly linked to family clusters, and almost all of them had epidemiological links to adult patients.^103^

Outside of China, pediatric COVID-19 data from Asia are limited at the time of writing these guidelines. To the best of our knowledge, there are no critically ill children in countries such as Japan and Singapore.

In India, children under 12 years of age constituted about 5.6% (1378 out of 24,586) of the total COVID-19 cases reported from the state of Tamil Nadu. In a limited screening of hospitalized children with severe acute respiratory illness, 0.5% were diagnosed with COVID-19.^104^ Critical illness is rare though a few deaths have been reported in young infants.

The Institute of Epidemiology Disease Control and Research in Bangladesh reports 102,292 confirmed COVID-19 cases in the country with 3% being children under 10 years of age, 7% children 11−20 years of age, and with this age group accounting for 2.3% of the total deaths in the country. No data on hospitalizations are available.^105^

### Europe

In Spain to date, of the 250,287 reported cases, 1399 children under 15 years of age (0.6%) have tested COVID-19 positive, and 52 (3.7%) of those children required PICU admission.^106^ No pediatric mortalities have occurred directly due to COVID-19, but in 3 infected children SARS-CoV-2 may have contributed to their death (Communication from Spanish Society of Pediatric Intensive Care, SECIP, Registry for SARS-CoV-2 as of April 21, 2020).

In Italy, at the time of manuscript submission, of the 162,004 reported cases, 1.7% are children 0−18 years of age. Among the pediatric patients, 18.2% were younger than 2 years of age and the 65.3% were older than 6 years. Only 6.3% of the pediatric patients required hospital admission with rare PICU admissions.^107^

In France, as of April 23, 556 pediatric patients (ages 0–14 years) have required hospitalization, 20 of which have required PICU admission, and 11 patients had underlying comorbidities. To date, five deaths (ages 0–19 years) have been reported.^108^

### North America

In North America, the Virtual PICU Systems (VPS) dashboard^109^ captures data for the United States and Canada from 183 PICUs. As of June 18, VPS reports 653 COVID-19-positive pediatric patients have required PICU admission and have in total required 4192 PICU days. The mortality rate is reported to be <1%. Of the patients requiring PICU admission, 15% of patients were under 2 years of age, 27% were 2–12 years old, and 37% were 12–18 years old with the remainder being adult patients admitted to the PICUs. Of the patients with data on discharge, 45% were previously healthy children with no comorbid conditions. Since the VPS data are self-reported by PICUs, there may be inaccuracies inherent to this dataset. Data from Mexico were not available.

### South America

Similar to other regions of the world, definitive pediatric data were difficult to identify. In South America, Brazil has been the most affected country to date, followed by Peru, Chile, Ecuador, Colombia, and Argentina. Data from Ministry of Health of Brazil report, as of April 27, 27 deaths from COVID-19 in pediatric patients (0–19 years old).^110^ The *Brazilian Research Network in Pediatric Intensive Care* (BRnet-PIC network) initiated a COVID-19 patients study and has enrolled 37 PICUs. As of May 27, this study included 32 confirmed pediatric critical care COVID-19 cases and no deaths. Data from Ministry of Health of Argentina report 2571 confirmed COVID-19 pediatric patients (0–19 years old), and one death as of May 24.^111^ The National Health Institute in Colombia (INS—*Instituto Nacional de Salud*) reports 2790 confirmed pediatric cases, 149 hospitalized patients, 31 PICU patients, and eight deaths as of May 31. The Peruvian Ministry of Health reports 4350 confirmed pediatric patients and 18 as of May 19.^112^

## Clinical trials in children

Multiple pediatric clinical trials evaluating COVID-19 in children are ongoing and can be identified at ClinicalTrials.gov^113^ with the search criteria COVID-19, Children, and selecting for “Child (birth-17)” under Eligibility Criteria. Among the 377 identified trails, 115 are interventional, 256 observational, and 43 are registries. Studies are being conducted in all regions of the planet; pediatricians and intensivists caring for children with COVID-19 and its sequalae are encouraged to identify regional studies and registries for enrolling patients. As this is a rapidly evolving disease entity, understanding the demographics, clinical presentation, diagnostic findings, biomarker profiles, and potentially effective treatments will be beneficial for the global community of health-care providers.

## Conclusion

This manuscript was developed to provide guidelines for managing COVID-19-infected critically ill pediatric patients in both high- and limited-resource settings. We also discuss the available regional pediatric data, which may be of particular interest given the limited pediatric COVID-19 data at present. SARS-CoV-2 has had a devastating impact on the adult population. Children comprise a small fraction of those impacted by this disease. We propose a set of initial guidelines to manage critically ill pediatric patients with COVID-19. These guidelines were based on the best available evidence, extrapolation of clinical evidence from adults, and the best practices where the evidence is lacking. As the COVID-19 pandemic evolves, implementing the proposed guidelines will help to reduce morbidity and mortality in pediatric patients. Updating these guidelines will be necessary based on additional data and the new evidence from ongoing clinical trials.
